# Institutional Costs of Guideline-Concordant Care Non-Receipt among Women with Breast Cancer

**DOI:** 10.1097/AS9.0000000000000489

**Published:** 2024-09-24

**Authors:** Kriyana P. Reddy, Oluwadamilola M. Fayanju, Stephany Perez-Rojas, Terry Hyslop, S. Yousuf Zafar, Justin Bekelman, E. Shelley Hwang

**Affiliations:** From the * Division of Breast Surgery, Department of Surgery, Perelman School of Medicine, The University of Pennsylvania, Philadelphia, PA; † Abramson Cancer Center, Penn Medicine, Philadelphia, PA; ‡ Penn Center for Cancer Care Innovation, Abramson Cancer Center, Penn Medicine, Philadelphia, PA; § Leonard Davis Institute of Health Economics, University of Pennsylvania, Philadelphia, PA; ∥ Division of Biostatistics, Department of Pharmacology, Physiology, and Cancer Biology, Thomas Jefferson University, Philadelphia, PA; ¶ Department of Medicine, Duke University School of Medicine, Durham, NC; # Department of Radiation Oncology, Perelman School of Medicine, The University of Pennsylvania, Philadelphia, PA; ** Duke Cancer Institute, Duke University, Durham, NC; †† Department of Surgery, Duke University Medical Center, Durham, NC.

## INTRODUCTION

Differences in rates of guideline-concordant care (GCC) receipt for breast cancer may contribute to persistent disparities in care and avoidable healthcare expenditures.^1,2^ GCC receipt is associated with reduced healthcare utilization and expenditures in women with breast cancer, which, at 14% of total cancer care costs, is the highest of any cancer in the United States (US).^3–6^ Prior work has documented high incremental costs incurred when treating advanced-stage *versus* early-stage breast cancer as well as recurrences.^7,8^ One previous study also found that concordance with National Comprehensive Cancer Network treatment guidelines for breast cancer drives significant reductions in total cost of care: patients with breast cancer who received GCC incurred 25–43% in cost savings *versus* patients who did not.^5^

Efforts to reduce breast cancer treatment costs may, therefore, benefit from improving GCC receipt. However, the financial costs to a health system arising from managing avoidable recurrences and cancer-related deaths among patients who do not receive guideline-concordant cancer treatment remain unknown. In this study, we sought to quantify the incremental, system-level costs of GCC nonreceipt among patients with breast cancer at an urban academic health system in the Northeastern United States.

## METHODS

We identified female patients ≥50 years with nonmetastatic hormone receptor-positive (HR+)/human epidermal growth factor receptor 2-positive (HER2+), cT2-4, and/or cN1-3 breast cancer receiving initial treatment at Penn Medicine between July 1, 2017, and December 31, 2020. We selected these clinical characteristics to have a cohort of patients who were potentially eligible for all forms of breast cancer treatment, that is, chemotherapy, endocrine therapy, anti-HER2 targeted therapy, surgery, and radiation therapy. For each patient, receipt or nonreceipt of neoadjuvant systemic therapy (NST, i.e., chemotherapy and anti-HER2 targeted therapy), lumpectomy followed by adjuvant radiation, mastectomy, adjuvant anti-HER2 therapy, and adjuvant endocrine therapy were identified. A patient was defined as receiving GCC if she followed 1 of 2 pathways: (1) NST followed by lumpectomy, adjuvant radiation, adjuvant anti-HER2 therapy, and adjuvant endocrine therapy or (2) NST followed by mastectomy, adjuvant anti-HER2 therapy, and adjuvant endocrine therapy. If a patient received ≥1 but not all of the treatments in a pathway, she was identified as not having received GCC. Counts and proportions of patients receiving and not receiving GCC were summarized in a flow diagram. Rates of recurrence were compared between those who did and did not receive GCC using a 2-sample test of proportions.

**FIGURE 1.**
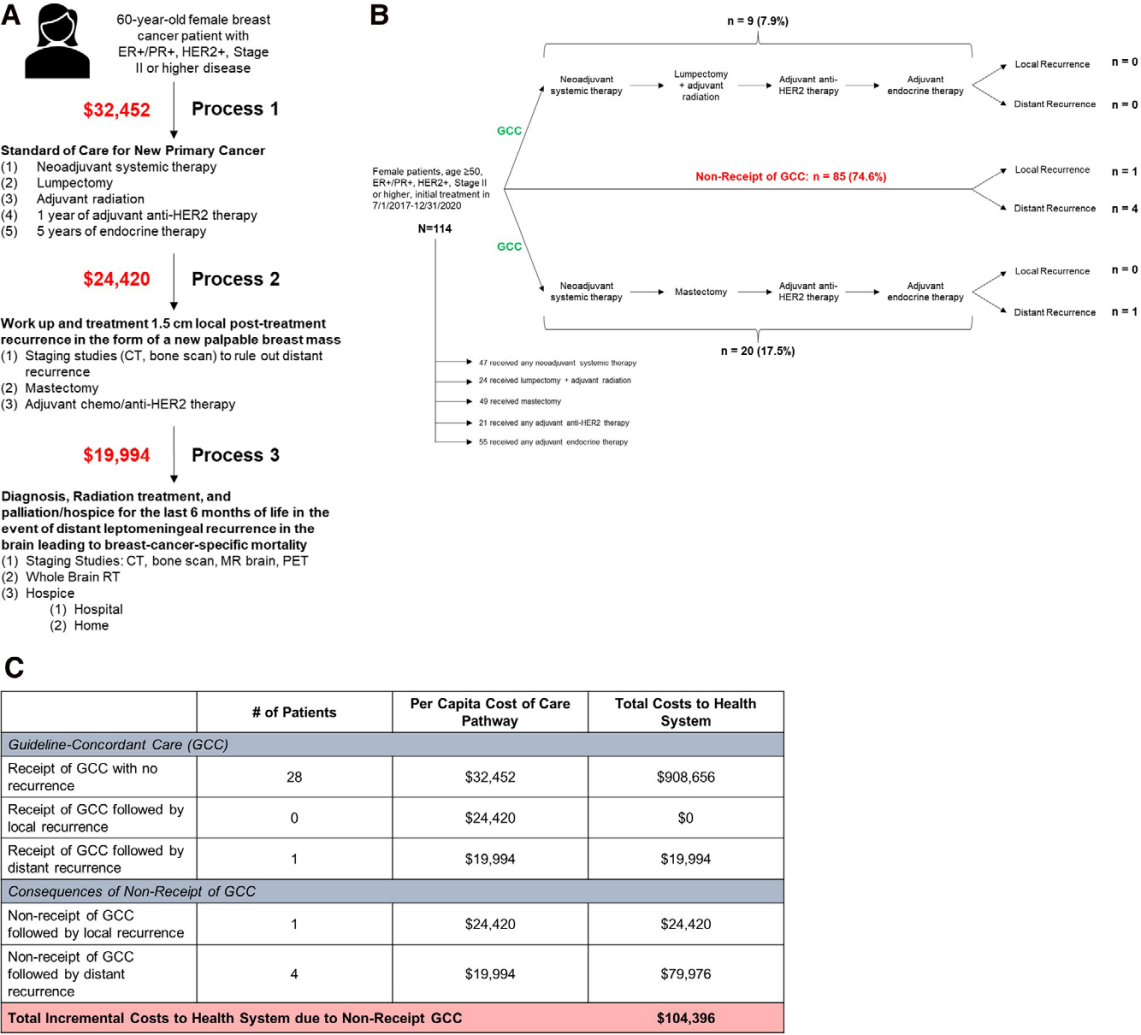
Processes and costs associated with guideline-concordant breast cancer care. A, Process map showing guideline-concordant care for 60-year-old female breast cancer patient with ER+/PR+, HER2+, Stage II or higher disease. Dollar amounts shown represent the cost of care associated with each process. B, Counts and proportions of patients receiving and not receiving guideline-concordant care (GCC) as well as patients having local and distant recurrences among patients meeting inclusion criteria in the Penn Tumor Registry. C, Total costs of care associated with receipt of GCC with or without subsequent recurrence, as well as nonreceipt of GCC with or without subsequent recurrence. The total incremental cost to the health system due to nonreceipt of GCC was calculated as the sum of costs of care arising from nonreceipt of GCC followed by local recurrence and costs of care arising from nonreceipt of GCC followed by distant recurrence.

In keeping with time-driven activity-based costing, which was recently conducted for breast cancer care by Nagra and colleagues,^9^ we assumed that personnel and the salaries they incur would be the primary drivers of cost. To estimate per capita costs of care for GCC as well as incremental costs for work-up and treatment of local and distant recurrences, we calculated standardized salary estimates for the health system averaged over the study period to adjust for the effects of the COVID-19 pandemic. Salary estimates were totaled to determine the total per capita cost to the health system for providing patient care in each of these pathways: (1) GCC for a new primary cancer; (2) work-up and treatment for a local recurrence in the form of a palpable breast mass; and (3) diagnosis, radiation treatment, and palliation/hospice for the final 6 months of life following leptomeningeal recurrence leading to breast-cancer-specific mortality (Fig. 1A). The number of patients with local recurrence following nonreceipt of GCC was multiplied by the per capita cost of care for local recurrence. The same was done for patients with nonreceipt of GCC followed by distant recurrence. These 2 figures were summed to determine the total incremental costs to the health system due to nonreceipt of GCC.

This study was deemed exempt from review by the Institutional Review Board of the University of Pennsylvania. Informed consent was not required as data were deidentified for analysis and reporting. All analyses were performed in R version 4.1.0 (R Foundation for Statistical Computing, Vienna, Austria). Statistical testing was 2-tailed with *P* < 0.05 deemed statistically significant. The study followed the Consolidated Health Economic Evaluation Reporting Standards.^10^

## RESULTS

We identified 114 patients meeting inclusion criteria. Of those patients, 47 (41.2%) received NST, 24 (21.1%) received lumpectomy and adjuvant radiation, 49 (43%) received mastectomy, 21 (18.4%) received adjuvant anti-HER2 therapy, and 55 (48.2%) received adjuvant endocrine therapy. Overall, 29 (25.4%) patients received GCC. The rate of recurrence among patients receiving GCC was 3.4% *versus* 5.9% among patients not receiving GCC (*P* = 0.61) (Fig. 1B).

The total cost of care to the health system for all patients receiving GCC and having no recurrences was $908,656. One patient received GCC and had a distant recurrence, representing a $19,994 incremental cost to the health system. The total incremental cost to the health system due to nonreceipt of GCC was $104,396, representing 11.5% of the costs to the health system for providing GCC to patients who ultimately did not have recurrences. Of the total incremental cost of non-receipt of GCC followed by recurrence, $24,420 (23.4%) was due to treatment and work-up for patients with local recurrences, and $79,976 (76.6%) was due to diagnosis, treatment, and palliation/hospice for distant recurrences (Fig. 1C).

## DISCUSSION

Among women potentially eligible for all forms of breast cancer treatment, only one-quarter received GCC, and management of recurrences in patients not receiving upfront GCC yielded an 11.5% incremental increase in total costs to the health system. These findings suggest that interventions aimed at improving the receipt of GCC may also improve the overall cost of breast cancer care delivery as well. To our knowledge, this is the first study to quantify the health system-level economic impact of GCC nonreceipt among patients with breast cancer.

Nonreceipt of GCC is associated with adverse effects including disease progression and recurrence, and differential receipt of GCC may be explained by factors such as structural racism, inequitable access to healthcare, and physician and patient preferences.^3,11–14^ Accordingly, improving rates of GCC receipt can mitigate both disparities and costs associated with treating potentially avoidable recurrences. Indeed, costs associated with breast cancer recurrences are estimated to contribute an additional $10,000 to $20,000 per patient to the overall cost of breast cancer care in the United States, which was roughly $30 billion in 2020^6,8^ Literature assessing the economic impact of GCC in breast cancer patients remains limited, but our findings suggest that interventions aimed at improving rates of GCC receipt may also reduce the total cost of care.

### Limitations

While GCC can also be defined to include duration of treatment (eg, 1 year of anti-HER2 therapy), this information was not an available measure in the data used for this study. Consequently, the estimated rate of receipt of GCC in this study may overestimate the true population rate of receipt. Second, this study relies on a small sample size. Third, the cost of care in this study did not include equipment, material, location, utility, medication, and other costs. Additionally, the data source lacked granular information on the actual costs of care for each patient, so we were unable to assess specific, itemized treatment-related costs incurred by the health system for each patient. Further work leveraging administrative claims data or patient-level cost data is warranted to validate the findings of this cohort study. Finally, we lacked data on specific reasons for the nonreceipt of GCC; additional work is needed to understand the mechanisms underlying GCC nonreceipt.

## CONCLUSIONS

In this study, we identified a 74.6% rate of GCC nonreceipt among women with locally advanced breast cancer who were potentially eligible for all forms of treatment. Among women not receiving GCC, 5.9% had local or distant recurrences, and costs associated with work-up and treatment of recurrences in patients not receiving upfront GCC amounted to an 11.5% incremental increase in total costs to the health system. The results of this study have broader implications for informing policy measures and interventions to mitigate differential rates of receipt of GCC for breast cancer and other areas within oncology.

## ACKNOWLEDGMENTS

We would like to thank Neil Crimins, Director, Service Line Analytics, Strategic Decision Support at Penn Medicine, for his help obtaining salary estimates to calculate cost of care in this study.
